# 40 years of the human T-cell leukemia virus: past, present, and future

**DOI:** 10.12688/f1000research.17479.1

**Published:** 2019-02-28

**Authors:** Yutaka Tagaya, Masao Matsuoka, Robert Gallo

**Affiliations:** 1 Institute of Human Virology, University of Maryland School of Medicine, Baltimore, MD, 21201, USA; 2 Department of Hematology, Rheumatology and Infectious Diseases, Faculty of Life Sciences, Kumamoto University, Kumamoto, 860-0811, Japan

**Keywords:** Human T-cell leukemia virus-1, human oncovirus, human retrovirus, adult T-cell leukemia, HAM/TSP, Central Australia, STD, vaccine

## Abstract

It has been nearly 40 years since human T-cell leukemia virus-1 (HTLV-1), the first oncogenic retrovirus in humans and the first demonstrable cause of cancer by an infectious agent, was discovered. Studies indicate that HTLV-1 is arguably one of the most carcinogenic agents to humans. In addition, HTLV-1 causes a diverse array of diseases, including myelopathy and immunodeficiency, which cause morbidity and mortality to many people in the world, including the indigenous population in Australia, a fact that was emphasized only recently. HTLV-1 can be transmitted by infected lymphocytes, from mother to child via breast feeding, by sex, by blood transfusion, and by organ transplant. Therefore, the prevention of HTLV-1 infection is possible but such action has been taken in only a limited part of the world. However, until now it has not been listed by the World Health Organization as a sexually transmitted organism nor, oddly, recognized as an oncogenic virus by the recent list of the National Cancer Institute/National Institutes of Health. Such underestimation of HTLV-1 by health agencies has led to a remarkable lack of funding supporting research and development of treatments and vaccines, causing HTLV-1 to remain a global threat. Nonetheless, there are emerging novel therapeutic and prevention strategies which will help people who have diseases caused by HTLV-1. In this review, we present a brief historic overview of the key events in HTLV-1 research, including its pivotal role in generating ideas of a retrovirus cause of AIDS and in several essential technologies applicable to the discovery of HIV and the unraveling of its genes and their function. This is followed by the status of HTLV-1 research and the preventive and therapeutic developments of today. We also discuss pending issues and remaining challenges to enable the eradication of HTLV-1 in the future.

## The discovery of HTLV-1

It has been 40 years since human T-cell leukemia virus (HTLV)-1 ^1^ was discovered by the Gallo group at the National Cancer Institute/National Institutes of Health (NCI/NIH) as the first human retrovirus. Thus, it is fitting to start this review by looking back at the chronology preceding the discovery of HTLV-1. The group, focusing on virology, has been pursuing the biochemical purification and characterization of the reverse transcriptase since the early ’70s. In the mid-’70s, they identified the first T-cell growth-promoting cytokine ^2^, now known as interleukin-2 (IL-2), in their efforts to enable a long-term culture of human T cells so they could study cells isolated from patients with leukemia. All of these efforts paved the way for the group to discover a new retrovirus from human T cells, the HTLV-1; subsequently, the same group discovered the second human retrovirus (HTLV-2) ^3^ and co-discovered the third human retrovirus, now known as HIV-1 as the cause of AIDS ^4^. In 1981, at a conference held in Kyoto (near Lake Biwa), the Gallo group reported that sera from patients with adult T-cell leukemia (ATL), described below, contain antibodies against the purified p24 protein of the HTLV ^5^. Later that year, a Japanese group led by Hinuma isolated a similar type C-retrovirus from an ATL cell line and also showed that sera from patients with ATL contain antibodies reactive with cells infected with the new virus ^6^. This virus was later shown to be identical to HTLV-1 ^7, 8^.

## Impact of HTLV-1 on the discovery of HIV

HTLV-1 was the first human retrovirus discovered ^9^. It shifted the paradigm in the virology world in many ways by demonstrating not only that retroviruses can infect humans but also that retroviruses can be pathogenic in humans. In 1982, the Gallo group reported the second human retrovirus, HTLV-2 ^10^. Around the same time, reports of a rare form of lung infection, *Pneumocystis carinii* pneumonia, among homosexual young men started to appear from the west-coast areas of the US and soon from other parts of the world, which the US Centers for Disease Control and Prevention named “acquired immunodeficiency syndrome” (AIDS). It drew the attention of virologists, and in 1982 Max Essex and Gallo postulated that a new type of (retro)virus may be associated with AIDS, although the scientific community remained skeptical. In 1983, the Montagnier group at the Pasteur Institute in Paris reported the discovery of a new retrovirus (lymphadenopathy virus, or LAV) from one patient with AIDS ^11^. In 1983 and 1984, the Gallo group reported the isolation of a human retrovirus (HTLV-III) in 48 patients with AIDS and, along with their blood test, linked the virus to AIDS as the cause ^4, 12– 18^. LAV and HTLV-III were shown to be the same virus ^19– 22^, and the name HIV (human immunodeficiency virus) was adopted in 1986. The technological approach was the same as for the HTLVs. Studies on HTLVs also provided both conceptual and scientific methodology critical to the discovery of HIV ^23^; thus, the discovery of HTLV-1 and -2 laid the foundation for the discovery of HIV. Moreover, it provided the framework by creating the “human retrovirology field”. Furthermore, the study on the *pX/Tax* gene of HTLV-1 accelerated the mechanistic understanding of the action of HIV through the study of regulatory elements of this virus ^24– 26^ (for example, *Tat* ^27^ and *Rev* ^28^), as *Tax* served as the prototypic example of human retroviral regulatory genes. HIV and HTLV can co-inhabit, as the first isolate of HTLV-III from the Gallo group came from a person who was doubly infected by HTLV-1 and HIV, most likely through blood transfusion, and the same T cells from this individual were producing HTLV-1 and HIV, which challenged the strongly held view at that time of “viral interference”, stating that a cell infected by a retrovirus resists superinfection by another retrovirus. This prevented the Gallo group from announcing the discovery of HTLV-III (HIV) for several months because of the confusion it caused to the group before they realized that these cultures contained HTLV-1 plus a new retrovirus (HIV). On the other hand, this established a new methodology of stably maintaining HIV in culture because CD4 T cells doubly infected by HTLV-1 and HIV would remain viable and keep producing HIV whereas an infection by HIV alone would kill the target CD4 T cells. This led to the concept that immortalized (transformed) CD4 T cells could continuously produce HIV. By adopting HIV to mature CD4 T cells already transformed by other causes than HTLV-1, the cells maintain their growth and enabled the mass production of HIV(as early as 1983), which was essential for establishing a global blood test for HIV ^4^ and central for the testing of anti-HIV drugs.

## Exceptional oncogenicity of HTLV-1

HTLV-1 was the first retrovirus identified from humans. Moreover, it is one of the first human viruses which were proven to have oncogenic effects in humans, together with Epstein–Barr virus (EBV) (also known as human herpesvirus-4) ^29, 30^ and papilloma viruses ^31, 32^. Although the oncogenic capability of papilloma viruses is as solid as that of HTLV-1, the extent to which EBV/HHV-4 directly causes malignancy is still in dispute ^30, 33^. Despite the prolific nature of EBV/HHV-4 reaching 90% prevalence among adult humans, the associated malignancies such as Burkitt’s lymphoma (BL), Hodgkin’s lymphoma and nasopharyngeal carcinoma are yet unconventional. In addition, BL can occur in the absence of EBV/HHV-4 ^30^. EBV/HHV-4 is the most powerful B-cell transforming factor *in vitro* ^30^. However, the pattern and regulation of EBV gene expression are strikingly different between *in vitro* transformed cells and *in vivo* BL cells ^34^. It is unquestionably a triggering factor for human malignancy, but EBV/HHV-4 appears to require co-factors to cause cancers ^30^.

HTLV-1 manifests many diseases, including ATL, HTLV-1–associated myelopathy/tropical spastic paraparesis (HAM/TSP), inflammatory disorders, especially uveitis, arthritis, and dermatitis, and an immune-deficient state, resulting in bronchiectasis that is causing high mortality in Central Northern Australia ^35^. (This topic will be elaborated on below, see also Table 1).

**Table 1.** List of human T-cell leukemia virus-1 (HTLV-1) diseases. HBZ, HTLV-1 bZIP factor.

Disorders	Disease manifestation	Prognosis, prevalence	Remarks	Therapy
Leukemia/Lymphoma	CD4 T-cell leukemia (adult T-cell leukemia, ATL)	Fatal leukemia with 4 subtypes Occurs in 3~5% of all carriers, in 20% after infection around birthtime	Develops after 3~5 decades of latency period.	No standard therapy
Myelopathy	HTLV-1 associatedMyelopathy/Spastic paraparesis, HAM/TSP.	Progressive, resembles multiple sclerosis Occurs in 0.3~5% of all carriers. More prevalent and rapidly progressive with infections associated with transplants.	Develops after 2~4 decades of latency period.	No standard therapy
Immunodeficiency	T-cell immunodeficiency Bronchiectasis	Seen even in asymptomatic carriers. Causing high morbidity/mortality among Australian indigenous people	More often seen with HTLV-1 subtype c (Melanesia)	No standard therapy
Inflammatory	Uveitis, arthropathy, dermatitis, exocrinopathy, myositis		Caused by HBZ?	

The major manifestation is the T-cell leukemia (ATL), and the lifetime risk of HTLV-1–infected people to develop ATL is estimated to be around 5%. However, a recent publication ^36^ from Taylor’s group suggests an intriguing re-estimation. Most ATL cases develop from those who contracted HTLV-1 at birth, through mother-to-child infection, but ATL is rarely seen in HTLV-1 carriers who have been infected in adulthood. This is an interesting contrast with the myelopathy HAM/TSP, the second major HTLV-1 disease, which is seen with higher prevalence and accelerated disease progression in those who were infected by HTLV-1 in adulthood because of a blood transfusion or organ-transplantation involving infected T-lymphocytes than those who were infected in the perinatal period. It is also known that perinatal infection accounts for about 20 to 25% of all HTLV-1 infections. Thus, the Taylor group estimates that the true risk of ATL among perinatally infected carriers could be as high as 25% ^36^, a striking number outweighing even the risk of lung cancer associated with tobacco smoking (around 16% ^37^). Earlier, we reported that HTLV-1 displays higher carcinogenic potential than that of any known human oncovirus. This new study suggests that HTLV-1 is arguably one of the most carcinogenic agents known to humans (but thankfully it is not easily transmitted from person to person). Another unique oncogenic nature of HTLV-1 is that it is a direct mechanism (see below for details). Because of the outstanding transforming capability of HTLV-1, we recently returned to its original name (human T-cell leukemia virus-1) and away from human T-lymphotropic virus-1. This was supported by a large majority vote at the 2017 HTLV-1 international meeting in Tokyo ^38^. (We are often asked whether an official committee needs to approve the naming of a virus. The only international committee in virology—the International Committee on Taxonomy of Viruses—clearly states on its home page that they deal only with taxonomy and that the name of individual viruses will be determined by a peer-reviewed publication. Thus, the recent renaming of the HTLV-1 to “leukemia virus” is legitimate and hopefully final.)

## Epidemiology of HTLV-1

**Figure 1.**
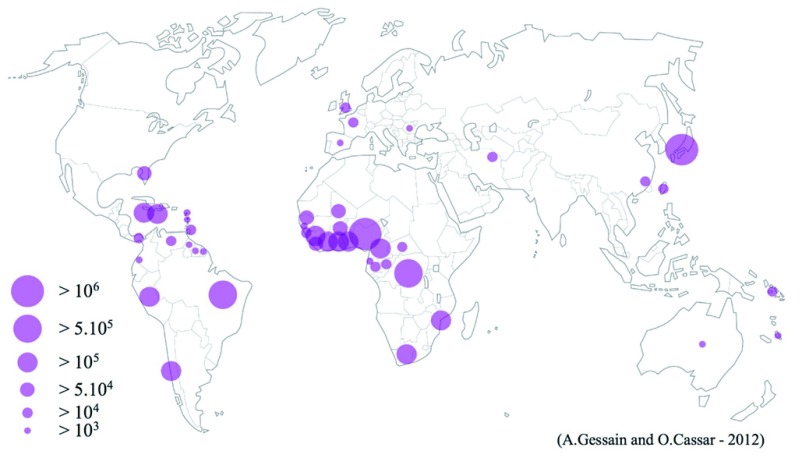
Global distribution of endemic foci of HTLV-1 infection. The figure represents an estimated number of HTLV-1–infected individuals, based on reliable epidemiological data from studies targeting pregnant women, blood donors, or different adult populations or a combination of these. It is noteworthy that, even within a given country, the endemic foci are not evenly distributed. Some of the “empty” areas represent those in which detailed epidemiological studies have never been conducted (for example, India and China). HTLV-1, human T-cell leukemia virus-1. Adapted from Gessain and Cassar ^40^ with their kind permission.

The discovery of ATL ^39^ predates that of HTLV-1 ^1^. The geographical distribution of patients with ATL, in Japan and in other parts of the world (for example, the Caribbean basin), prompted researchers to hypothesize that an infectious microorganism causes the disease. As shown in Figure 1 (kindly permitted by the authors ^40^ and the publisher for adoption here), HTLV-1 shows a unique endemic distribution on a global scale, involving Japan, the Caribbean islands, South America (Brazil, Colombia, Chili, and Peru), West and Central Africa, Romania, parts of the Middle East (especially Iran), and Central Australia. As discussed below, HTLV-1 is unusually inactive in replication as a virus and rather exists as a genetic element in infected cells after integration. This behavior of HTLV-1 has been used for anthropology studies as well ^41^ to track historical migration of humans. For example, the high prevalence of Asian type HTLV-1 in Japan and among the indigenous people of North and South America suggests that the migration of HTLV-1 (subtype B) infected humans from Asia to America when these two parts were connected through land bridge during the last ice age almost 10,000 years ago. The map, however, also suggests that global epidemiology studies are still to be completed. For example, some heavily populated areas such as India, China, much of Russia, and Sub-Saharan Africa do not offer detailed epidemiologic information on HTLV-1 infection. The strange lack of appropriate funding for such studies is a common obstacle on a global scale for HTLV research.

HTLV-1 can be transmitted from carrier mother to the baby mainly through breast feeding. Equally important is the horizontal transmission by sex and transfer of infected lymphocytes, such as blood transfusion and organ transplant. It is important to emphasize that HTLV-1 infection requires absolute cell-to-cell contact ^42^ but that cell-free virions generally do not cause infection. Shortly after the discovery of HTLV-1, many countries adopted HTLV-1 screening in donated blood, effectively reducing the risk of HTLV-1 transmission by transfusion. However, such screening is not yet performed all over the world. Moreover, only few countries (the UK and France) perform HTLV-1 screening on individuals who donated organs. The US has dropped similar screening because of the concerns over losing intact organ sources ^43^ and the argument that HTLV-1 infection in adulthood should not be a medical threat because the prevalence of ATL is low and requires a latency period of four to five decades ( https://optn.transplant.hrsa.gov/resources/guidance/guidance-for-htlv-1-screening-and-confirmation-in-potential-donors-and-reporting-potential-htlv-1-infection/). Recent studies in Japan, however, provide a different view on this. Yamano and co-workers have studied the post-transplantation development of HAM/TSP by infected organs, which suggests that the myelopathy occurs at about 20 times higher risk (nearly 10% of post-transplantation over 0.3% of mother-to-child transmission). Additionally, the disease rapidly progresses to morbidity within 5 years of infection, which is a striking contrast to the two to three decades of delayed onset and additional two decades of disease development for reaching the final stage of HAM after perinatal infection. This new study ( https://www.mhlw.go.jp/file/04-Houdouhappyou-10905500-Kenkoukyoku-Zoukiishokushitsu/0000068136.pdf) may be a serious alert to the global medical community to review and revise their policy on HTLV-1’s threat as a blood-borne pathogen.

## An emerging issue: endemic in Central Australia

One of the most recent additions to the HTLV-1 epidemiology is Central Australia. Early in the ’90s, HTLV-1 infection in Melanesia was observed. Furthermore, Melanesian HTLV-1 is genetically divergent from major subtypes found in other parts of the world ^44^ (cosmopolitan subtype A seen in Japan, the Caribbean, Central and South America, the Middle East, and North and South Africa and the Central African subtype B) and hence named subtype C. Like subtype A and B, subtype C appears to have evolved from simian T-cell leukemia viruses (STLVs) (the ancestral viruses to HTLV-1/2), but intriguingly genetic analysis suggests that the evolution of subtype C might have been independent of other subtypes ^45^. Recent studies revealed that the divergence is highest in a part of the *pX* region near the 3’ end of the virus. This region encodes for small HTLV-1 proteins such as p30, p12, p13, and p8 that have been extensively studied by the Franchini group (NCI), which showed that these proteins expressed from the open reading frame (ORF) I or II display diverse regulatory functions ^46^. How this would affect the pathogenesis of subtype C is still under investigation.

The profound impact of HTLV-1 on the indigenous people of Australia was not recognized until recently, which is another testimony that our understanding of global HTLV-1 infection is still incomplete. Einsiedel *et al*. demonstrated that some communities of Aboriginal people showed 45% HTLV-1 sero-positivity ^47^. The Central Australian HTLV-1 infection presents two emerging issues for the HTLV-1 community. One is the opportunity to study differences of new subtype C and other subtypes and connect them to its pathogenicity. As described below, HTLV-1 subtype C may be distinct from major subtypes in terms of the diseases it causes. In addition, as discussed below, it is a challenge because solving this issue requires not just biological research and medical treatment but social support as well. Again, the threat of HTLV-1 to humankind has been severely underestimated. So it is good news that the Australian government has announced new funding to address the issue.

## Pathogenic mechanism of HTLV-1 diseases

The disease concept of ATL was established in the mid-’70s by Takatsuki and co-workers ^39, 48^ before HTLV-1 was discovered; then another HTLV-1 disease was identified around 1985. A unique myelopathy called TSP was described in the Caribbean area ^49^. Gessain and de Thé demonstrated that HTLV-1 infection is associated with this neuronal disease ^50^. In 1986, Osame *et al*. showed that HTLV-1–infected individuals develop a unique myelopathy (HAM) ^51^. The two diseases are identical and hence the name HAM/TSP was adopted. Later studies show that HTLV-1 is also associated with inflammatory diseases ^52, 53^ other than the two diseases already mentioned, such as uveitis ^54, 55^, arthropathy, pneumopathy, dermatitis, exocrinopathy, and myositis. Furthermore, HTLV-1 may cause immunodeficiency. Clinicians earlier observed that ATL is often associated with immunodeficiency which may cause opportunistic infections, but even asymptomatic carriers (ASCs) have mild immunodeficiency (K. Takatsuki, personal communication). This is being revisited from a new perspective in light of the recent finding that infection of HTLV-1 subtype C among Australian indigenous people is causing high morbidity/mortality by triggering chronic lung infections, including bronchitis, bronchiolitis, and bronchiectasis ^56^.

Globally, ATL is the major manifestation of HTLV-1. ATL is now categorized into four types: chronic ^57^, smoldering ^58^, acute ^48^, and lymphoma ^59^ (reviewed by Takatsuki ^60^). The direct causative role of HTLV-1 for this leukemia is solid. Shortly after the discovery of the virus, the *in vitro* transforming ability of this virus was reported ^61^. The transforming ability of HTLV-1 was subsequently mapped to a region between the *pol* and 3’ long terminal repeat (LTR), named the *pX* region, and a non-canonical oncogenic component was identified and named Tax-1. Tax-1 does not show sequence homology to any mammalian gene including known oncogenes. Tax-1 strongly activates the transcription of many host genes ^62– 64^ in addition to HTLV-1’s 5’LTR ^65^. Soon, it was shown that the transcriptional activation by Tax-1 is mediated by nuclear factors, including nuclear factor kappa B (NFκB) ^66, 67^. Subsequent studies depicted how Tax-1 activates the NFκB pathway ^68^. Tax binds to IKKγ (inhibitor of NFκB kinase gamma) and activates the IKKα/β/γ complex ^69– 71^, which facilitates the nuclear translocation of p50/p60 of NFκB. Tax also enhances the processing of NFκB p100 into the active p52 form and thereby activates the “non-canonical” NFκB pathway ^68^. Alternatively, TaxBP1, a cellular binding protein to Tax which functions as an autophagy receptor, is known to repress TRAF-6 (TNF receptor-associated factor-6)/NFκB signaling. Tax can restore NFκB signaling by binding to TaxBP1 and modulating its function.

To validate the transforming role of Tax *in vivo*, Tax transgenic mice were generated. Using the 5’ LTR of HTLV-1 as enhancer/promoter, Tax expression caused mesenchymal tumors in mice ^72^. Another study demonstrated that Tax transformation is mediated by the downstream activation of NFκB ^73^. More recently, Bazarbachi and co-workers showed that Tax transforms Drosophila eye cells ^74^ and Cheng *et al*. showed that Tax immortalizes human dendritic cells *ex vivo* ^75^. Thus, Tax has broad transforming effects in a variety of cells across species. However, the development of T-cell malignancy by Tax requires the T cell–specific expression of this gene (Hasegawa and Hall *et al*. ^76^). A small caveat of this study was that the malignant cells do not express CD4 or CD8; thus, they resemble pre–T cell leukemia/lymphoma whereas *in vivo* ATL cells show a mature regulatory T (Treg)-like phenotype, although expression of CD4 may not be found in all tumor cells of patients with ATL ^77^. These studies show that Tax is oncogenic but does not determine cell specificity of ATL.

**Figure 2.**
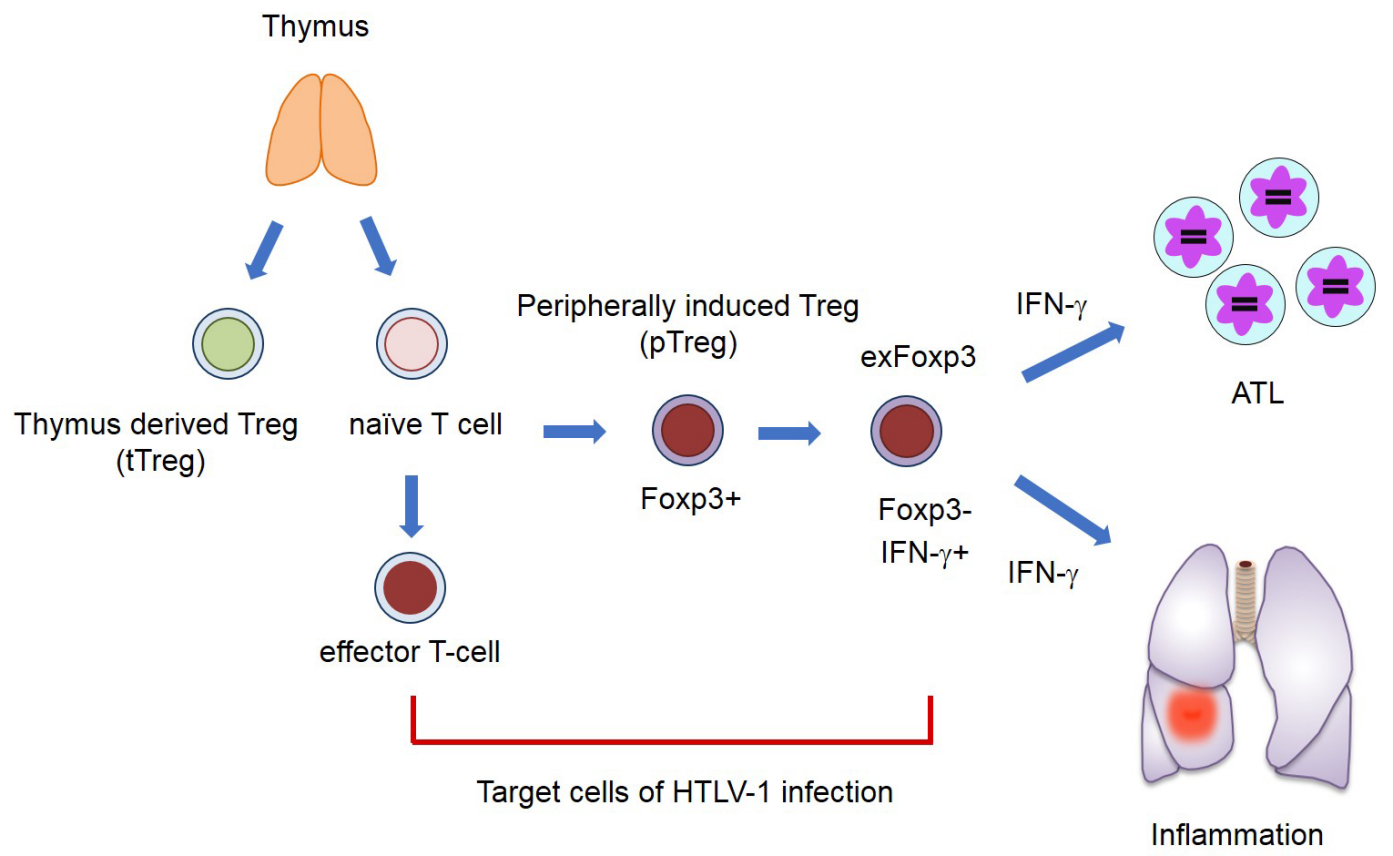
Inflammatory phenotype of HTLV-1–infected cells triggered by HBZ. Target cells of HTLV-1 are effector/memory T cells and Foxp3 ^+^ T cells *in vivo*. HBZ promotes the transcription of the *Foxp3* gene by enhancing transforming growth factor-beta (TGF-β)/Smad signaling, which leads to the peripheral increase of induced Tregs (iTregs) and Foxp3 ^+^ T cells. However, note that Foxp3 expression is unstable in these cells. When Foxp3 expression is lost, “exFoxp3” cells produce IFNγ, which causes inflammation and promotes leukemogenesis. HBZ, human T-cell leukemia virus-1 bZIP factor; HTLV-1, human T-cell leukemia virus-1; IFNγ, interferon gamma; Treg, regulatory T.

However, a completely independent scenario was presented through studies conducted recently. In 2002, Gaudray and Mesnard *et al*. discovered that HTLV-1’s minus strand contains a reading frame which encodes a potential transcription factor named HBZ (HTLV-1 bZIP factor) ^78^. Subsequent studies by a group led by one of the authors (MM) demonstrated that HBZ is a very complex gene with many intriguing facets ^79– 83^. It appears to function not only as a protein but also as a regulatory RNA which promotes proliferation of host cells ^84^. As a protein, HBZ activates the transforming growth factor beta (TGF-β)/Smad pathway, which leads to transcriptional activation of the *Foxp3* gene ^85^. This finding explains why *in vivo* ATL leukemic cells are Foxp3-positive and HTLV-1–infected cells in ASCs and patients with HAM/TSP are within the Foxp3 ^+^ subpopulation of CD4 T cells ( Figure 2). It is noteworthy that there is no evidence that Tregs are the only exclusive target of HTLV-1 infection ^86^. Instead, HTLV-1 orchestrates the T-cell molecular program to transform conventional T cells to look like Tregs ^87, 88^. Another important message is that the inflammatory responses seen with HTLV-1 infections can be caused by HBZ ^89^, in part by the production of various pro-inflammatory cytokines, including TGF-β ^85^ and interferon gamma (IFNγ) ^90^, by infected CD4 T cells and non-infected blood cells. Additionally, HBZ inhibits canonical NFκB and Wnt pathways ^91^. HBZ also induces expression of CCR4 and TIGIT at the cell surface ^92, 93^. Importantly, CCR4 is implicated not only in migration but also in the proliferation of T cells ^94^. Likewise, the upregulation of TIGIT, a co-inhibitory molecule, may help evade anti-ATL/HTLV-1 immunity and facilitate the propagation of ATL leukemic cells ^92^. Finally, evidence that HBZ can also cause CD4 T-cell malignancy was provided when this gene was expressed as a transgene in mouse T cells ^87^. Curiously, malignant T cells in HBZ transgenic mice express markers (for example, CD25 and CCR4) which are commonly seen with *in vivo* ATL cells. So what is the indispensable transforming component of HTLV-1? At the moment, this remains an open question. Earlier studies revealed that HTLV-1 is quite often (>40%) integrated as a defective provirus which still later helps in the development of ATL ^95^. In many cases, the deletion involves the 5’ LTR and cripples the capacity of the virus to express components in the positive strand including Tax. Such defective viruses are much more commonly seen among patients with ATL than in ASCs ^96^. Curiously, no deletion of 3’ LTR was found, suggesting the critical need for HTLV-1 to retain an antisense-encoded gene (or genes). Also, recent analyses using the next-generation sequencing technology demonstrate that the majority of the HTLV-1 transcripts in *in vivo* leukemic cells are driven by the 3’LTR, but not by the 5’LTR (see below for more). Antisense genes appear to be a common and critical element of retroviridae. Bovine leukemia virus (BLV), HTLV-1’s sibling virus in cows, also expresses antisense transcripts which are expressed in bovine tumors and associated with cellular transformation ^97^. HIV-1 also encodes an antisense gene and uses it to mediate critical functions for the virus ^98^. So we ask the question again, Tax or HBZ? Curiously, HTLV-1’s sister virus HTLV-2 shows no oncogenic capability, although HTLV-2 also encodes a *Tax* gene ( *Tax-2*), which is a potent transforming gene *in vitro* ^99^. Whereas *Tax-1* and *-2* are highly conserved (>89% identity) ^17^, the antisense genes (HBZ and APH-2 of HTLV-2) are very divergent. The Green lab has shown that APH-2 of HTLV-2 functions as a regulatory factor in host cells ^100, 101^ and is functionally very different from HBZ. These results favor HBZ as the dominant factor in the leukemic transformation. However, the activation of NFκB, which has been considered an essential part of HTLV-1-related cellular transformation, is only caused by Tax, but not by HBZ ^93^, which is seen as a caveat of “the HBZ hypothesis”. Two recent studies indicate that Tax expression *in vivo* is transient and seen as only a burst during the cell cycle ^102, 103^. The pulsive expression of Tax, however brief, facilitates the survival of HTLV-1–infected cells by minimizing the alert to the host immune surveillance mechanism ^103^ since the Tax protein *per se* is strongly immunogenic ^104, 105^. Moreover, host restriction factors play a role in silencing the function of Tax and viral replication ^106– 108^ and conceals the role Tax might have played in the early phase of cellular transformation. Similar to many other cancer cells, HTLV-1–inserted/transformed cells accumulate somatic mutations. The advancement of next-generation sequencing technology has accelerated the research addressing host genomic alterations caused by HTLV-1. An integrated molecular analysis ^109^ (genome, exome, transcriptome, methylation, and so on) highlighted that alterations were frequently found in Tax-interactome and T-cell activation pathways, including NFκB signaling, trafficking, and immunosurveillance pathways. Gain-of-function mutations were also seen in association with various T-cell signaling molecules, observations that are consistent with previous consensus on the importance of the Tax-NFκB axis in the cellular transformation by HTLV-1. Curiously, this study also recapitulated the above-mentioned reports ^95, 96^ that integrated HTLV-1 proviral genome often displays deletions in the 5’ viral segments, resulting in defective viral replication and production. Moreover, viral transcripts are produced predominantly from the antisense strand, consistent with the importance of HBZ in the development and maintenance of established ATL cells. It is also of note that most antisense transcripts extend beyond the 5’ LTR of HTLV-1 and include neighboring host genes juxtaposed to the integration site ^109^, suggesting the novel role for 3′ LTR as the driver of HTLV-1–related transcriptome. It has been held that the integration of HTLV-1 into the host genome is random. However, new studies challenge this paradigm ^110, 111^. Most recently, Rosewick and van den Broeke *et al*. showed that integration of HTLV-1 and BLV preferentially occurs near cancer drivers and that insertional mutagenesis appears to drive the clonal persistence and expansion of transformed T cells ^112^. All of these findings were supported by another recent publication ^113^. In addition, Satou and Bangham *et al*. ^114^ demonstrated that HTLV-1 has a binding site for CTCF, a regulator of chromatin structure. They showed evidence that the insertion of HTLV-1 creates a novel loop structure between the provirus and host genome by recruiting CTCF, which naturally alters the expression of proviral and host genes. Collectively, the characterization of HTLV-1 integration and its impact on the host transcriptional and epigenetic regulations constitute an emerging research field, as is the role of Tax and HBZ for cellular transformation. Even the once-accepted paradigm of the necessity of NFκB activation, may need to be further investigated in light of these new findings.

Whereas the oncogenic process of ATL appears mainly molecular and virologic, the pathogenesis of HAM/TSP is more immunologic ^115^. In HAM/TSP, the major symptoms appear to be caused by the damages to spinal neuronal cells by inflammatory T cells. The suggested mechanism includes the acquisition of an inflammatory nature by HTLV-1–infected CD4 T cells and non-leukemic CD8 T cells. As discussed earlier, HBZ of HTLV-1 facilitates the generation of inflammatory CD4 T cells ^85, 89, 91^. Possibly relevant to the pathogenesis of HAM/TSP is the recent observation that the HBZ protein localizes exclusively in the cytoplasm of lymphocytes in patients with HAM/TSP ^116^, as opposed to the predominant nuclear localization of HBZ in leukemic cells in patients with ATL, suggesting the presence of a HAM/TSP specific use of HBZ in the development of the disease. In addition to cytokines, chemokines CXCL-9 and -10 are produced by HTLV-1–infected CD4 T cells and play important roles in recruiting inflammatory T cells to the site of neuronal destruction ^117^. Furthermore, Azimi, Jacobson, and Waldmann have suggested that γc-cytokines, in particular IL-2 and IL-15, are produced by HTLV-1–infected CD4 T cells via the transcriptional trans-activation by Tax and in turn activate CD8 T cells into inflammatory T cells ^118– 121^ which produce tissue-damaging cytokines such as IFNγ. These molecules are attractive therapeutic targets in treating HAM/TSP, as discussed below.

## Therapy development for HTLV-1 diseases

As mentioned earlier, the prognosis for HTLV-1 diseases is still poor despite almost 40 years of studies on mechanisms and efforts to develop therapeutic strategies. The acute/lymphomatous ATL is very resistant to conventional chemo/radiation therapy. Thus, surface markers specific for HTLV-1–infected CD4 T cells were sought after to enable targeting. The first marker was IL-2Rα/CD25 ^122^ (then called the Tac antigen ^123^). Anti-IL-2Rα/Tac therapy, including the conjugation of the antibody to toxins or α/β-emitters, has been tried with some success ^124– 127^, but resistant leukemic clones quickly emerged. More recently, CCR4, a chemokine receptor, was identified as an ATL-specific marker ^94, 128– 132^ and anti-CCR4 therapy (using a defucosylated anti-CCR4 antibody) has been clinically tested in Japan with promising results ^129, 133– 136^. The only caveat was that anti-CCR4Ab (mogamulizumab) also depleted natural regulatory T cells (nTregs) and caused hyper-autoimmunity (Stevens–Johnson syndrome) in some cases, which led to one fatal case in the treatment ^137^. Nevertheless, it has been approved as an anti-ATL drug in Japan and clinical trials are being performed in the US as well. Arsenic trioxide, which has been used for AML treatment, was also shown to have anti-ATL effects ^138, 139^ and showed therapeutic effects on chronic ATL cases ^140^, and there is suggestive evidence that this treatment might eradicate “leukemia-initiating cells” ^141^. Also, anti-viral drugs have been tested. Previous findings suggest that HTLV-1 does not actively replicate in CD4 T cells in carriers and patients, prompting the field to consider that anti-viral therapy is not an option in treating HTLV-1 diseases. However, recent research presents more diversified views on this topic ^142– 146^ as anti-viral treatments show some promising results in clinical trials. Collectively, it is fair to say that promising treatments are developing for ATL, but additional strategies should be encouraged as well, including T cell–mediated immunotherapies targeting HTLV-1 components. Historically, it has been repeatedly shown that Tax of HTLV-1 is highly immunogenic and that HTLV-1–infected individuals have anti-Tax Ab and anti-Tax T cells in their circulation, which prompted the field to explore anti-Tax strategies. However, Asquith and Bangham *et al*. ^147^ investigated which epitope of HTLV-1 would elicit T-cell immunity leading to decreased proviral load of HTLV-1. Surprisingly, it was not anti-Tax or anti-Env but anti-HBZ immune responses which correlated with protective effects ^147^. Additional work by Sugata and Matsuoka *et al*. appears to solidify this possibility ^148^ and offers a promising new venue for the future. As in many other leukemias, bone marrow transplantation (BMT) is being used. Curiously, attempts of autologous BMT were unsuccessful ^149^. In contrast, allogeneic BMT resulted in complete remission in some cases, and it has been suggested that the extent of graft-versus-host response correlates with a favorable outcome ^150^. Immune cells in patients with ATL may be tolerized against HTLV-1, most likely because their immune system has developed in the presence of HTLV-1.

Compared with the pathogenesis of ATL, the pathogenesis of HAM/TSP is more complicated as the damage of spinal cord neuronal cells, the core event causing myelopathy, is mediated by inflammatory responses involving CD8 T cells triggered by HTLV-1–infected CD4 T cells in a bystander manner ^151^. So strategies targeting both CD4 and CD8 could be pursued. Here again, anti-CCR4 therapy, which should help eliminate HTLV-1–infected CD4 T cells ^152^, was recently tried with intriguing results in a clinical trial in Japan ^153^. First, the treatment showed quick improvements of clinical symptoms, prompting the field that the presumed low replication of HTLV-1 *in vivo* may need reinvestigation. Anti-CCR4 strategy was among the first to show therapeutic efficacy, so the completion of the clinical trial, including the monitoring of long-term effects, is keenly awaited. As mentioned above, CD8 T cells play as important a role as CD4 T cells in HAM/TSP, so the CD8 targeted therapy can be pursued as well. Studies from NIH labs (Waldmann and Jacobson) demonstrated that one unique characteristic of T cells from patients with HAM/TSP is that they grow *ex vivo* without any exogenous stimulation (for example, antigen or cytokine), which they named spontaneous proliferation ^118^, which presumably reflects the constitutive activation of T cells in patients with HAM/TSP. This is because HTLV-1 infection causes CD4 T cells to produce T-cell growth factors such as IL-2 and IL-15 ^118, 120, 121, 154, 155^. Thus, inhibition of these cytokines by antibodies ^121^, Jak inhibitors ^156^, or newly described multi-cytokine inhibitory peptide (BNZ132-1or BNZ-1 ^154, 155^) synthesized by one of the authors (YT) can subside the T-cell activation in patients with HAM/TSP and is a viable option for treating HAM/TSP for which no established therapy is yet available. In summary, recent years have seen more promising developments for HTLV-1 diseases. The time has come for more testing of novel ideas to cure or reduce morbidity from these diseases.

## Prevention and eradication of HTLV-1: the need for a global coordination of HTLV-1 research and its implementation

As mentioned above, HTLV-1 is among the most carcinogenic agents known to humans and has the additional burden of causing other diseases. Yet owing to its extremely weak dissemination and to its ancient infection of humans (giving time for humans to adapt), HTLV-1 is not considered to be as much of a global threat as other viruses. However, we have seen an instantaneous burst of virulence with other viruses due to mutations (for example, influenza virus, Ebola, and HIV), so we should not be so sure. Moreover, in several parts of the world, it is not as uncommon as previously noted. The modes of HTLV-1 transmission and replication suggest that the infection is preventable. Recently, we wrote an open letter to the World Health Organization (WHO), urging them to pay due awareness for HTLV-1, as we strongly feel that HTLV-1’s threat is vastly under-recognized ^157^; for example, it was not on the WHO’s list of sexually transmitted diseases, and remarkably in recent years under Director Harold Varmus (who was a chicken retrovirologist and molecular biologist), the NCI did not even have it on the list of human tumor viruses. We argued that the mother-to-child transmission can be controlled by screening and educating HTLV-1 carrier pregnant women. Three decades ago, a prefecture in Japan (Nagasaki) started such an endeavor, which reduced the HTLV-1 prevalence in the area from 7.2 to 1.4% ^158^. It is now adopted by the Japanese government into a nationwide anti-HTLV-1 strategy. The horizontal transmission of HTLV-1, by sex and transfer of infected blood cells, is equally preventable. To this end, some countries have initiated HTLV-1 screening in donated blood in the late ’80s. However, blood screening is not yet implemented in any nation in Africa, one of the most highly endemic regions of HTLV-1 infection. An additional emerging concern is the HTLV-1 infection upon organ transplantation which may cause increased development of HAM/TSP with rapid progression (described above). As of now, very few nations (only France and the UK) are conducting HTLV-1 screening in donated organs. Attempts to create funding to facilitate the screening as well as invention of new technologies to make such screening easier and attainable are still needed.

*What about a vaccine?* From the late ’80s to early ’90s, several groups came out with an HTLV-1 (and STLV) vaccination plan, which was effective but only partially so ^159– 161^. However, our knowledge of HTLV-1 biology, together with the maturation of the vaccination field in general, has vastly advanced since then. The Franchini group (NCI) has used more efficient adjuvants, and the vaccine was proven effective in macaques ^162^. Recent studies suggest that targeting HBZ, a new strategy, may be more effective than conventional strategies ^147, 148, 163^. However, development of anti-HTLV-1 therapeutic/preventive vaccination is severely hampered by the lack of funding for clinical trials.

As the conclusion of this review, we argue, on behalf of the global HTLV-1 community, that efforts toward the eradication of HTLV-1 constitute a global health need. At the ninth annual meeting of the Global Virus Network in Melbourne, Australia in September 2017, we met representatives of the Aboriginal community who pleaded to be rescued from their sufferings from HTLV-1. We urge medical/health agencies all over the world that the threat of HTLV-1 is real and there are still unmet medical needs to be resolved and much more awareness is needed. Finally, progress in developing a vaccine for HTLV-1 should be easier than HIV because it has less variation, and may provide important lessons for HIV.
